# A new adaptive videogame for training attention and executive functions: design principles and initial validation

**DOI:** 10.3389/fpsyg.2014.00409

**Published:** 2014-05-13

**Authors:** Veronica Montani, Michele De Filippo De Grazia, Marco Zorzi

**Affiliations:** ^1^Department of General Psychology, University of PadovaPadova, Italy; ^2^Center for Cognitive Neuroscience, University of PadovaPadova, Italy; ^3^IRCCS San Camillo Neurorehabilitation HospitalVenice Lido, Italy

**Keywords:** videogames, attention, attention deficits, executive functions, cognitive enhancement

## Abstract

A growing body of evidence suggests that action videogames could enhance a variety of cognitive skills and more specifically attention skills. The aim of this study was to develop a novel adaptive videogame to support the rehabilitation of the most common consequences of traumatic brain injury (TBI), that is the impairment of attention and executive functions. TBI patients can be affected by psychomotor slowness and by difficulties in dealing with distraction, maintain a cognitive set for a long time, processing different simultaneously presented stimuli, and planning purposeful behavior. Accordingly, we designed a videogame that was specifically conceived to activate those functions. Playing involves visuospatial planning and selective attention, active maintenance of the cognitive set representing the goal, and error monitoring. Moreover, different game trials require to alternate between two tasks (i.e., task switching) or to perform the two tasks simultaneously (i.e., divided attention/dual-tasking). The videogame is controlled by a multidimensional adaptive algorithm that calibrates task difficulty on-line based on a model of user performance that is updated on a trial-by-trial basis. We report simulations of user performance designed to test the adaptive game as well as a validation study with healthy participants engaged in a training protocol. The results confirmed the involvement of the cognitive abilities that the game is supposed to enhance and suggested that training improved attentional control during play.

## INTRODUCTION

Cognitive enhancement through videogame playing is a hot topic in cognitive science. Most of the literature on the effect of videogame play is centred on “action” videogames, which are remarkably challenging in terms of visual and attention demands. Indeed, many investigations have focused on the modulation of visual skills and have revealed that videogame players (VGPs) outperform non-videogame players (NVGPs) on a variety of visuo-attentional tasks (Green and Bavelier, 2003, 2006a; for reviews see Spence and Feng, 2010; Boot et al., 2011; Hubert-Wallander et al., 2011a; Latham et al., 2013). For example, VGPs showed to be better in localizing the target in many different visual search tasks (e.g., Castel et al., 2005; West et al., 2008; Hubert-Wallander et al., 2011b), they were better in suppressing irrelevant information (e.g., Mishra et al., 2011; Wu et al., 2012) and in general they showed to have more available attentional resources (e.g., Green and Bavelier, 2003, 2006b; Dye et al., 2009a).

Nevertheless, there is also evidence that videogame playing enhances a variety of other cognitive skills (Green and Bavelier, 2003; Dye et al., 2009a; Anguera et al., 2013) and that cognitive processes different from visuo-spatial ability might benefit from playing more strategic games (e.g., Basak et al., 2008). For example, Colzato et al. (2010) reported that VGPs suffer smaller task switching cost than NVGPs, suggesting that they have better cognitive control (see also Cain et al., 2012; Strobach et al., 2012). Karle et al. (2010) suggested that the smaller switch cost is the consequence of more efficient task reconfiguration due to a superior ability to control attentional resources (also see Meiran et al., 2000).

Action videogame playing also seems adequate for training executive control skills that are crucial for the coordination of different tasks in complex situations. For example, Strobach et al. (2012) showed that VGPs outperformed NVGPs in a dual task condition (but see Donohue et al., 2012, for contrasting results) and, even more convincingly, that non-gamers trained with an action videogame suffered less dual-task cost after training in comparison to non-gamers trained with a puzzle game. It is worth nothing that the latter result was confirmed in the study of Chiappe et al. (2013) using a more complex task that was shown to predict performance in real-life settings.

Selective and controlled aspects of attention appear to benefit more of videogame playing relative to transient, automatic aspects (Chisholm et al., 2010). Clark et al. (2011) suggested that better performance of VGPs is explained by an improvement in higher-level abilities such as attentional control, in addition to better bottom-up visual processing. Accordingly, a neuroimaging study confirmed lesser recruitment of the network associated with the control of top-down attention in VGPs, despite their superior performance in a visual search task relative to NVGPs (Bavelier et al., 2011). This result was interpreted as evidence that VGPs are more efficient in the allocation of attention.

Studies comparing VGPs and NVGPs on many different tasks invariably show that VGPs are faster across a wide range of tasks and they do not show speed-accuracy trade-offs (Dye et al., 2009b; but see Nelson and Strachan, 2009). Moreover, videogame training was shown to be a helpful training regimen for providing a marked increase in speed of information processing in elderly (Drew and Waters, 1986; Clark et al., 1987; Anguera et al., 2013).

It is worth noting that most of these studies do not establish a causal link between videogame play and cognitive enhancement because they do not control for pre-existing differences between VGPs and NVGPs (Kristjánsson, 2013). However, some studies have compared the performance of two groups of non-players before and after a different type of training. For example, an action videogame was compared to a game that made heavy demands on visuomotor coordination but, unlike action video games, did not require the participant to process multiple objects at once at a fast pace. Action-trained participants showed greater training-induced improvements than participants trained on a control game, thereby showing that the benefits of play are trainable to a non-game player population (Green and Bavelier, 2003, 2006a,b; Feng et al., 2007; Strobach et al., 2012). There is also some evidence that learning/enhancement is not specific to the trained task but there is some degree of generalization to untrained aspects (Green and Bavelier, 2006b; Mathewson et al., 2012) and some transfer to a completely different and more “ecological” domain (Gopher et al., 1994; Rosenberg et al., 2005; see Boot et al., 2011, for a critical discussion).

The aim of the present study was to develop a novel adaptive videogame for training attention and executive functions, with particular emphasis on design features that make the game suitable for brain-damaged patients as a tool to support cognitive rehabilitation. Despite some contrasting findings (Boot et al., 2008; Murphy and Spencer, 2009; Irons et al., 2011), videogames seem to enhance a variety of cognitive skills and they appear to be a promising tool to train cognitive abilities (e.g., Achtman et al., 2008; Basak et al., 2008; Anguera et al., 2013; Franceschini et al., 2013). Moreover, neuroplasticity in the adult brain could be guided with specific training to yield better recovery (e.g., Krainik et al., 2004; Gehring et al., 2008). The rationale for designing a new videogame, despite the great variety of commercial videogames that are currently available, was twofold. First, designing a novel videogame allows the inclusion of specific features in a theory-driven manner as well as to implement a fine control of the difficulty dimensions, including trial-by-trial adaptation to user performance. Second, the graphical user interface of commercial videogames might be too demanding for patients with cognitive deficits in terms of speed, visual complexity, or motor requirements.

Before presenting the videogame, we start with a discussion of the theoretical principles that guided our design choices in terms of structure and features of the game. We then report a modeling study in which we simulated users with different abilities to assess the efficiency of the adaptive algorithm in estimating the “performance space” of the user, which is crucial for the online adjustment of game difficulty. Finally, we validated the game with unimpaired participants (healthy young adults) to ensure that the game involves the activation of the desired cognitive functions as well as to assess the effect of a short training period (<10 h over 2 weeks). Note that the evaluation of videogame training for the rehabilitation of brain damaged patients is left to a future clinical trial.

## GAME DESIGN PRINCIPLES

Dysexecutive syndrome and attention deficits are common consequences of traumatic brain injury (hereafter TBI; e.g., Levine et al., 1998; Stuss and Levine, 2002). Indeed, the acceleration-deceleration mechanism of traumatic injury implies that the frontal and temporal lobes are the most frequent damaged sites, with subsequent impairment of a wide range of high-level functions (Povlishock and Katz, 2005). The resulting impairments in attention and executive functions can profoundly affect an individual’s everyday cognition, with difficulties in the management of very simple daily activities (Sohlberg and Mateer, 2001). Attention deficits have been found to be significantly correlated with the inability to return to work (Van Zomeren and Brouwer, 1985; Vikki et al., 1994). Because of the related disabilities and the increasing number of people suffering from this pathology, the development of effective rehabilitation strategies should be considered of high priority. Furthermore, the recent finding of Kamke et al. (2012) that increased visual attention demands entail a decrease in motor cortex plasticity strongly supports the notion that attention can be a potent modulator of cortical plasticity.

The design of the game was guided by principles relevant for the rehabilitation of cognitive deficits in TBI patients. The first principle was to enhance mental flexibility, which is the ability to respond to environmental changes in an efficient way. Mental flexibility implies efficient deployment of attentional resources accordingly with the context, as to select and maintain the cognitive set that is appropriate for the current goal. In order to increase mental flexibility, training should engage patients in switching between different cognitive sets. The alternation of different tasks requires reconfiguration of the new task and inhibition of the current active set, that is the set of the previous task (Monsell, 2003). Switching can be predictable or unpredictable (e.g., Andreadis and Quinlan, 2010). If the tasks alternate in a predictable way, participants can take benefit of the information about the switch and consequently prepare the switch endogenously. If the tasks alternate in a random way (i.e., unpredictable switch), switching task requires a faster reconfiguration of the mental set that is exogenously triggered by the task itself. Overall, unpredictable switching is considered more demanding than predictable switching but since TBI patients seem to have problems in the endogenous engagement of attention (Stablum et al., 1994) as well as slow information-processing speed (e.g., Mathias and Wheaton, 2007), they can benefit from training with both types of switching. Therefore, training should initially involve predictable switching and then progress to unpredictable switching.

Patients have also problems with managing two simultaneous tasks (Sohlberg and Mateer, 2001). The multitasking deficit can be ascribed to their slower processing speed (Dell’Acqua et al., 2006; Foley et al., 2010) or to a specific impairment in the ability to divide attention (Serino et al., 2006). There is evidence that dual task training improves the ability to divide attention by speeding up information processing through the bottleneck in the prefrontal cortex (Dux et al., 2009). Finally, increasing attentional load induced by multitasking has been shown to hinder visuo-spatial monitoring in patients with right hemisphere stroke (Bonato et al., 2010, 2012, 2013). Regardless of the specific mechanism underlying the deficit, extensive training with dual tasking can greatly reduce multitasking cost (Van Selst et al., 1999; Schumacher et al., 2001; Tombu and Jolicoeur, 2004). Therefore, a second important principle that should guide the design of game training is to improve the ability to achieve different goals at the same time. Dual-tasking requires to maintain the cognitive sets of both the tasks, dividing attentional resources between the two goals.

Including both tasks switching and dual-tasking within the training may be considered as a reflection of the complexity of daily living. In a more ecological environment, the individual has often to manage with situations that require to quickly change the goals or to pursue two goals simultaneously. Flexible or integrated training regimens, requiring constant switching of processing and continuous adjustments to new task demands have also been claimed to lead to greater transfer (Bherer et al., 2005).

The third principle that should guide the design of a game for cognitive training is to stimulate planning ability. Indeed, disorganized behavior of TBI patients is another aspect of their poor ability to control cognitive resources. They are not able to maintain the intentions in goal directed behavior, likely because the sustained attention system is compromised. This results in a high level of distractibility and a cue-dependent behavior (Levine et al., 2011). Flexibility in planning and strategy selection should be promoted by trial-by-trial changes of the game playground, thereby requiring the gamer to manage a novel situation every time. This implies that achieving the goal would require to choose the adequate strategy, with the interruption of automatic responses and monitoring of the performance, accordingly with the task. Consequently, the gamer would need to plan the correct sequence of actions to achieve the goal and to actively maintain this set of actions.

Patients’ performance tends to be more variable and less consistent over time in comparison to healthy controls (Stuss et al., 1989, 1994). A critical challenge is to organize the progression of practice in a way that promotes performance improvement while finding a balance between patient variability and the choice of optimal task difficulty. Moreover, TBI patients are often unaware of their impairments (Prigatano and Schacter, 1991) and their anosognosia is a further challenge because rehabilitation can be seriously hindered by the lack of patient cooperation. Anosognosia predicts recovery from stroke (Gialanella and Mattioli, 1992) and experience-dependent plastic reorganization requires attention to be paid to the activity in question (Recanzone et al., 1993). Therefore, an important principle is to maintain attention and motivation providing sufficient positive reinforcement. Videogames are a useful tool because they are more entertaining than other training programs but in order to maximize the benefit they should be equipped with an adaptive algorithm. Motivation for playing can be maintained by programming the algorithm to adapt the difficulty of the game to a level that is challenging but feasible, for example by keeping the probability of success around 0.75. The ability to complete the task gives a “reward” to the gamer that may enhance his/her motivation. Moreover, the adaptive difficulty is an important aspect in enhancing training effects (Holmes et al., 2009; Brehmer et al., 2012).

Finally, every task should be completed in a pre-determined amount of time, accordingly with the difficulty of the task. The time pressure acts to encourage speeding up of processing, as consistently shown in the literature on videogame playing (Dye et al., 2009b; Hubert-Wallander et al., 2011a).

## THE GAME: “LABYRINTH”

### OVERALL GAME DESIGN

A little man moves along a maze to reach a goal. The game character is controlled by the gamer through a joystick. The walls that form the maze are variable: both their quantity and their location change at every trial accordingly with the task difficulty. The only constraint in the random distribution of the walls is that the software avoids the appearance of closed areas because this may prevent goal achievement.

**FIGURE 1:**
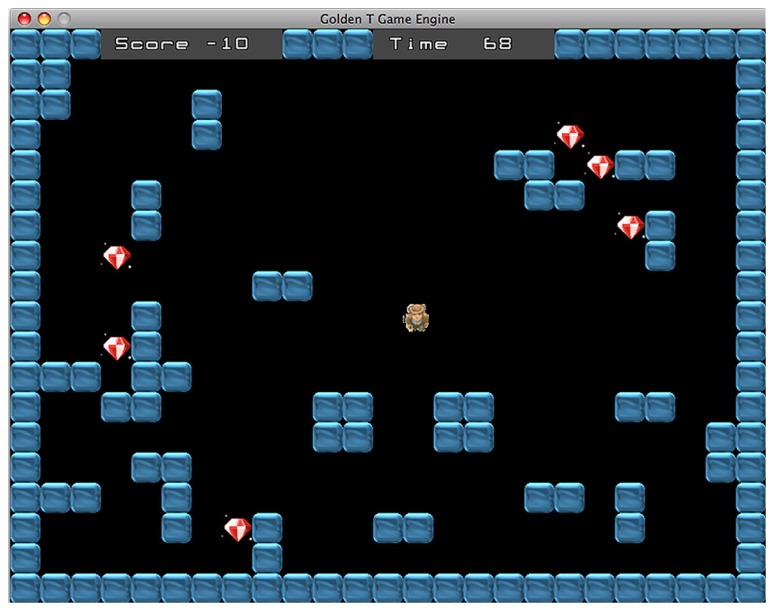
**Diamond task.** The goal is to collect all diamonds within the time limit.

**FIGURE 2:**
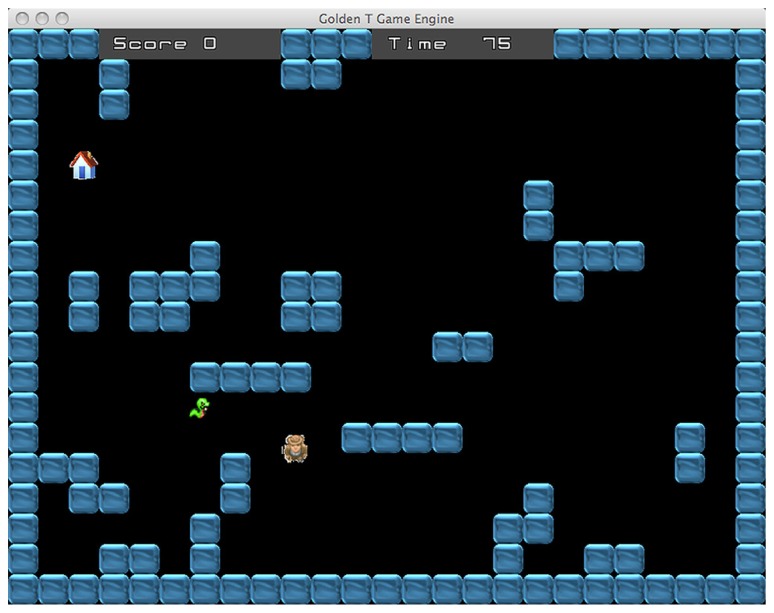
**Snake task.** The goal is to avoid to be caught by the snake and reach a “shelter” house that appears at a random location.

The maze difficulty changes accordingly with the type of task. Indeed, the game includes two different tasks, the “Diamond Task” (hereafter DT) and the “Snake Task” (hereafter ST). Overall, every task has eight difficulty levels, across a continuum ranging from the less demanding (level 1) to the more demanding (level 8). In the DT (see **Figure 1**), the easiest maze is the one with as few walls as possible and the number of walls increases in conjunction with the improvement of performance. Conversely, in the ST (see **Figure 2**), the easiest maze is the one with as many walls as possible and accordingly, the number of walls decreases with the improvement of performance.

The goal of the game character depends on the nature of the current task. In the DT, the man has to collect the diamonds that are randomly distributed across the play area. The DT resembles the open-ended version of the Travelling Salesman Problem (TSP), a task that strongly involves planning and is also representative of many real-world situations (Cutini et al., 2008). Given a set of spatial locations represented by points on a map, the task consists in finding an itinerary that visits each point exactly once, ensuring that total traveled distance is as short as possible. While the classic TSP requires to return to the starting point, the open-ended version introduces a distinction between start- and end-point so that participants have to perform an open path instead of a loop. TPS can be solved with multiple close-to-optimal solutions and usually healthy participants change strategy during the pathway to optimize performance. Therefore, the task achievement requires controlling and modifying the plan accordingly with the evaluation of both the current position and the remaining path. Basso et al. (2001) showed that TBI patients tend to use a fixed strategy until the end of the task without considering the alternative options, consistent with the hypothesis that TBI patients are unable to inhibit the current strategy in order to chose a better one (also see Cutini et al., 2008, for a computational model of normal and impaired performance in the TSP). In the DT, the number of diamonds ranges from one, in the less demanding level, to eight in the more demanding level. The achievement of the goal requires the participant to plan a route that allows to collect every diamond within the time limit. Usually the best overall strategy is to follow the shortest path passing through the diamonds.

In the ST, the man has to avoid to be caught by a snake and to reach a “shelter” house that appears at a random location (see **Figure 2**). The range of difficulty is enforced by controlling the running speed of the snake, as well as the time limit for trial completion. The achievement of this task requires a very different strategy compared to the diamond task. The best strategy is sometimes just the opposite: indeed, if the man takes the shortest way to arrive at the shelter house, it is likely that the snake will catch him. Avoiding to be caught often requires to choose a longer route, sometimes moving even in the direction opposite to the house location. Likewise, depending on the location of the house and the disposition of the maze walls, another good strategy may be to stop for a while, in a strategic location, waiting for the snake to take a wrong route. In this way, reaching the house becomes possible provided that the gamer chooses the right timing and moves quickly. Basically, the task requires “to trick” the snake. Therefore, accomplishment of the tasks requires adopting complex strategies involving the ability to plan and sometimes also inhibiting the most “automatic” action.

**FIGURE 3:**
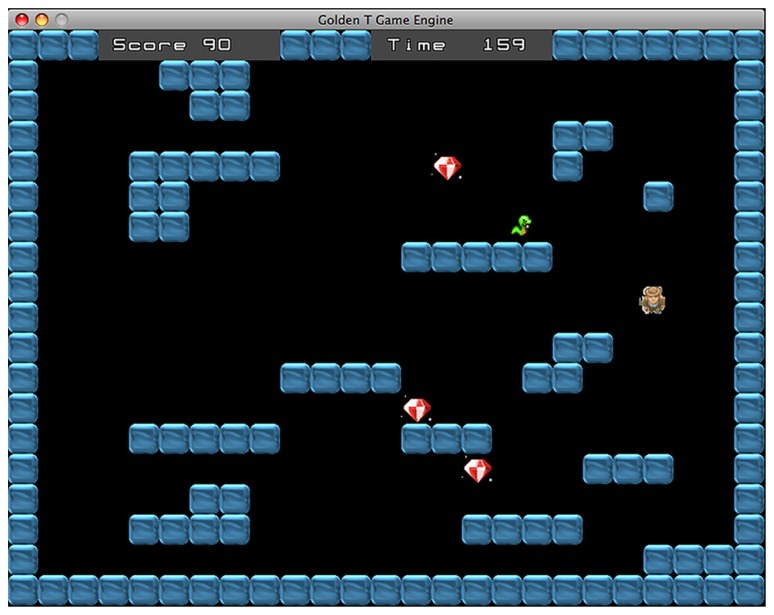
**Dual task.** In these trials the goal is to avoid the snake and collect the diamonds at the same time. The shelter house appears only when all diamonds have been collected.

The DT and ST alternate between each other with a frequency that is adjusted according to the performance score. The difficulty of this “switch condition” has four levels ranging from a completely predictable switching, when one task follows the other, to a completely random switch. The two medium levels involve a switch every two trials and a switch every three trials, respectively. In some trials, the gamer has to perform the two tasks simultaneously (see **Figure 3**). In these trials the participant has to avoid the snake and to collect the diamonds at the same time. Contrary to the standard ST, in this case the shelter house appears only after all diamonds are collected. Overall, the successful performance requires reaching two simultaneous goals: collecting every diamond and avoiding the snake within the time limit. The dual task condition is administered only if the percentage of success is higher than 60%. When the gamer achieves this performance level, the probability to receive a dual task trial is 30%. In this way, the participant can reach enough expertise in the two single tasks before managing the more difficult dual task condition. If the trial is performed correctly the player receives some points, whereas if the participant fails to reach the goal some points are subtracted from the score. Every six trials the gamer receives a feedback concerning his/her performance.

### ADAPTIVE DIMENSIONS

Following Wilson et al. (2006) we used a multidimensional learning algorithm for continuous, online adaptation of task difficulty to the current performance of the gamer. Adaptation was implemented using three dimensions of difficulty:

(1) Time limit: the time limit to perform the task. The level of difficulty is ranging from 5 to 100 s. It is updated every trial.
(2) Task difficulty: overall it has eight levels but the difficulty depends on the task. In the DT it is related to the number of diamonds that have to be collected (from one to eight), while in the ST it is related to the snake speed. In both tasks the difficulty consists also in the number of walls of the maze (see Overall Game Design). It is updated every trial.
(3) Switch condition: the type of switch, predictable vs. unpredictable. It has four levels (every trial, every two, every three, random). This dimension is updated every 12 trials.

The combination of the three dimensions forms the “training space.” This can be described as a cube with the three dimensions of difficulty as sides (Wilson et al., 2006). Every trial corresponds to a point within this cube (with the coordinates defined by the values of the three difficulty dimensions) and every point is associated with a certain probability of success. Higher probability is associated with easy trials and the opposite for the hard trials. Each user will be associated with a different probability of success matrix that defines the individual “performance space”. For example, a patient who is more impaired in inhibiting automatic responses and less impaired with speed of processing will have a higher probability of success in the “time” dimension and lower probability of success in the “task difficulty” dimension.

**FIGURE 4:**
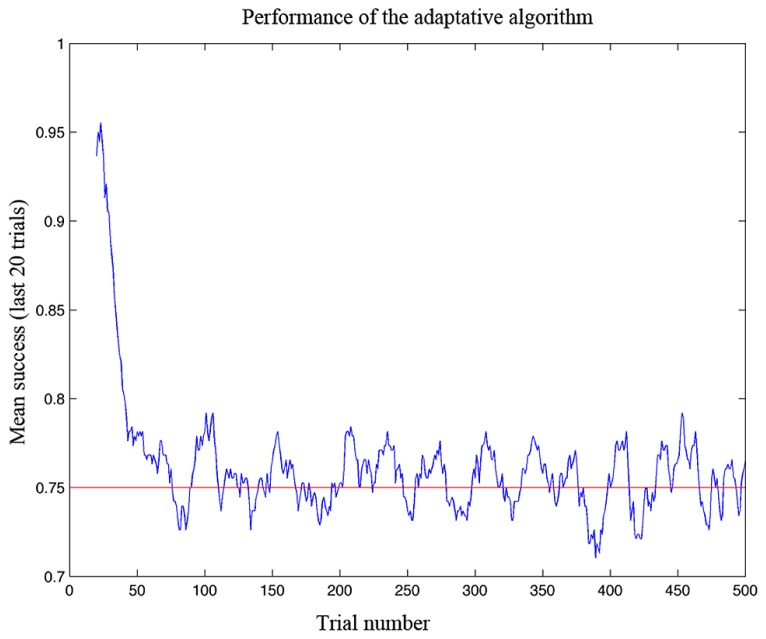
**Performance of the adaptive algorithm in ensuring a defined level of success in simulation testing.** The graph shows the gamer’s success rate (measured as a running average over the last 20 trials) as a function of trial number. Note that the algorithm adapted to the ability of the gamer in less than 100 trials and then kept the success rate at the desired level of 75%.

The task of the algorithm is to estimate the performance space of the user accordingly with the current performance. After sampling points within the training space, the algorithm uses the responses of the player to build an interpolated model of the entire performance space. Then, it selects a random point in the space which it estimates to correspond to the level required to maintain performance at 75% of accuracy (**Figure 4**). Moreover, with the game advancing, the algorithm updates the performance space accordingly with the success or failure of the gamer.

### SIMULATION

In order to test the algorithm, performance in the game was simulated with a Matlab model (http://www.mathworks.co.uk/). The simulator represented the performance space of the gamer at a given moment by a matrix of the success probability, as in the adaptive algorithm. The subject’s performance space was characterized by a “performance threshold,” that is the set of coordinates which specified the high success zone (in which the probability of success is 100%). Outside the high success zone, the probability of success for a given type of game trial was calculated by determining the distance between its location and the subject threshold and applying a sigmoid function to this distance (Wilson et al., 2006). If the trial location is far from the threshold the probability to be successful at this level of difficulty will be low or zero, whereas if the trial location is close to the threshold the probability to be successful will be high. The “performance threshold” could move up simulating the improvement of performance as a consequence of the training. In the simulator, learning rate (LR) was assumed to be a function of the derivative of the sigmoid (Wilson et al., 2006). For example, if the gamer has a successful performance in a trial far away from the threshold, her performance has a fast LR.

**FIGURE 5:**
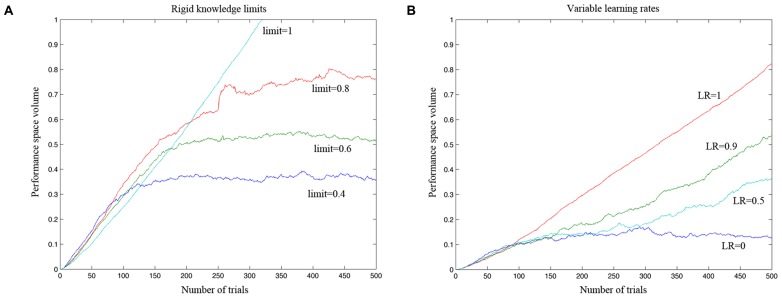
**Simulation testing the efficacy of the adaptive algorithm to accurately estimate a model of the gamer ability. (A)** The estimated performance space of a virtual gamer who has a fixed level of performance and zero learning rate. The four virtual gamers have different performance limits, ranging from 1 (which implies 100% probability of success in the entire performance space) to 0.4 (which implies 100% probability of success in 40% of the performance space). After 100 trials, the algorithm could estimate fairly well the performance space of the gamer as defined by the simulator and it could clearly distinguish between different gamers with different levels of performance. **(B)** Simulation carried out to test if the algorithm can distinguish between gamers with different levels of learning rate (LR). The algorithm was able to adjust the rate of increase in difficulty as a function of the learning rate of the different simulated gamers.

**FIGURE 6:**
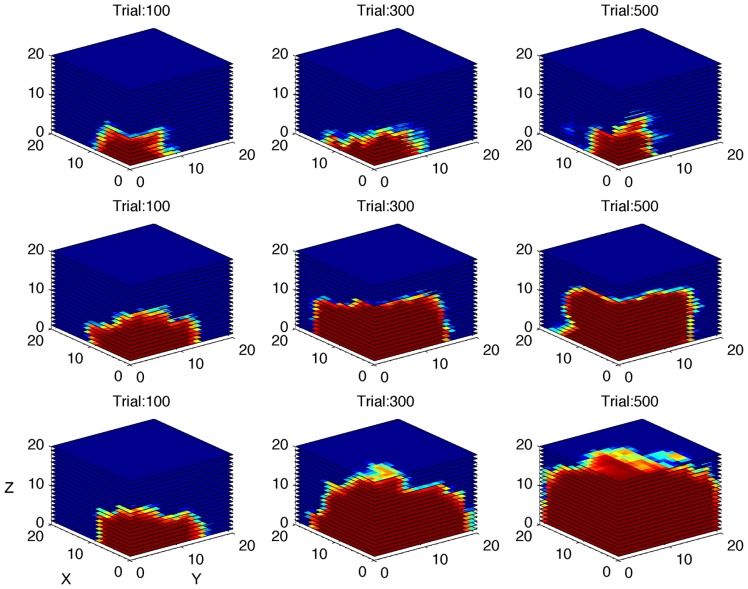
**Simulation of gamers with different performance limits.** Performance space estimated by the algorithm after 100, 300, and 500 simulated trials, shown as three-dimensional cube, for three different virtual gamers with fixed limit of performance and zero learning rate (top row: limit = 0.4; middle row: limit = 0.6; bottom row: limit = 0.8). The red area represents high probability of success.

The first simulation was carried out with a virtual gamer who has a fixed level of performance and zero LR. The aim of the simulation was to test if the algorithm was able to develop an accurate model of the gamer ability. In **Figure 5A**, the ability of the algorithm to estimate the performance of four different gamers is represented on a trial by trial basis. At the beginning of the game the algorithm cannot reliably estimate the different performance spaces. After 100 trials, the estimates diverge and then reach the specific level of performance corresponding to the fixed limit set for each simulated gamer. **Figure 6** shows a tridimensional representation of the performance space of three different virtual gamers (with fixed limit of performance).

**FIGURE 7:**
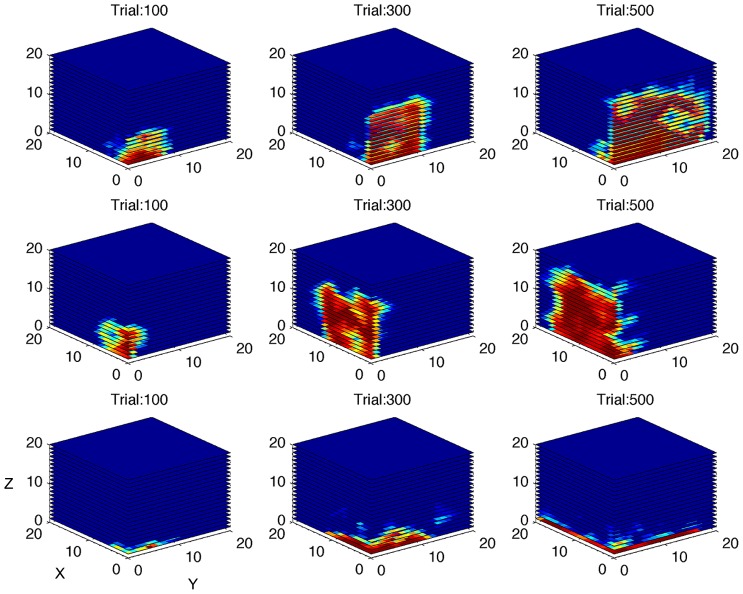
**Simulation of gamers with different learning rates.** Performance space estimated by the algorithm after 100, 300, and 500 simulated trials, shown as three-dimensional cube, for three different virtual gamers with null initial performance space and different learning rate (LR) for the three dimensions (top row: LR = 0 for X dimension and LR = 1 for Y and Z dimensions; middle row: LR = 0 for Y dimension and LR = 1 for X and Z dimensions; bottom row: LR = 0 for Z dimension and LR = 1 for X and Y dimensions). The red area represents high probability of success. Note that the performance space does not expand through the dimension with zero learning rate.

The second simulation investigated the algorithm’s ability to distinguish between gamers with different levels of LR (**Figure 5B**). The performance of the gamer with zero LR does not change over time. Conversely, the slope of the performance of the gamers with higher LRs becomes steeper accordingly with the rate of increase. As shown in **Figure 5B**, the algorithm was able to adjust the rate of increase in difficulty as a function of the LR of the different simulated gamers. **Figure 7** shows the performance space of three different gamers, with different LRs for the three dimensions (i.e., time limit, task difficulty and switch condition). For each gamer, LR for one dimension was set to zero (i.e., the gamer does not learn at all) and the LRs for the other two dimensions were set to 1 (i.e., the gamer learns quickly). It is possible to appreciate how the estimate of the algorithm changes accordingly with the characteristic of the gamer. The probability of success expands rapidly for the two dimensions with high LR, whereas it does not change for the dimension with zero LR.

## VALIDATION OF THE GAME WITH UNIMPAIRED PARTICIPANTS

The videogame “Labyrinth” has been conceived as a tool for training specific skills. The goal of the validation study was to test the new videogame with unimpaired participants. A group of healthy young adults was engaged in a training protocol which involved daily 40 min play sessions with the videogame for 2 weeks.

We also sought to establish that the game practice involves the targeted abilities by evaluating the presence of the dual task effect and the task switching effect in the different dependent measures of the game during the first play session. If the alternation between DT and ST works as switch condition we should observe a cost in the participants’ performance when one task is followed by the other task relative to when it is followed by the same task (Monsell, 2003). Usually the cost consists in worse accuracy in the new task relative to the repeated one and/or in slower RTs in the new task relative to the repeated one. Likewise, performing the two tasks at the same time should be more difficult than performing a single task, thereby revealing the cost of multi-tasking.

Videogame output is quite different from that of classic experimental paradigms based on choice reaction times. We extracted three different performance measures from the videogame that became the dependent variables of our analyses. The three types of score were:

(1) Success rate: whether the task was completed with success or not, within the time limit;
(2) Overall time: the time taken to complete the task;
(3) Diamond Time (DT): the time to collect the first diamond;

The DT measure is closer to the trial onset than the other two measures and collecting the first diamond is clearly an immediate and objective sub-goal of the task. Therefore, it should be more sensitive in uncovering effects that might be otherwise undetectable.

Note that the first two measures cannot be used to evaluate the effect of training across sessions because the adaptive algorithm keeps the performance level around 75% by continuously changing the different adaptive dimensions. Nevertheless, we assessed the participants’ progress across sessions in terms of task difficulty level and time limit (see Adaptive Dimensions above). We predicted a trend toward increasing difficulty level and decreasing time limit across sessions as a marker of improved performance in the videogame during training. Moreover, we assessed the effect of training on dual tasking and task switching performance using the DT measure, because the latter is not influenced by the choices of the adaptive algorithm. The time taken to collect the first diamond was compared between single and dual-task conditions (i.e., dual task cost), as well as between repeated and new task conditions (i.e., task switching cost). We expected a decrease of both costs across training sessions.

### METHOD

#### Participants

Twenty undergraduate students from the University of Padua participated in the study. Their mean age was 20.8 with range of 19–25 years. They had normal or corrected-to-normal vision.

#### Apparatus, stimuli, and procedure

The videogame “Labyrinth” was installed on the personal computer of each participant. Given that the participants were healthy young adults, we set lower bounds for the level of difficulty (level 3) and the time limit (25 s). The training period was 14 days long. Participants played with the game for 40 min everyday. The duration of the daily training session was enforced by self-termination of the game. The individual performance space estimated by the adaptive algorithm (see Adaptive Dimensions) was saved at the end of the session and reloaded at the beginning of the next session. This ensured that the difficulty of the game was immediately restored to the level achieved in the previous play session. Total play time across the 14 sessions was 9 h and 30 min.

### RESULTS

First, we analyzed the data collected in the first session of game playing. The aim of this analysis was to assess the presence of the dual task effect and the switch effect. We performed analysis of variance with the type of task as within-subjects factor. The game performance trend across the training sessions was analyzed using mixed-effects multiple regression models (Baayen et al., 2008). Data were analyzed in the R environment (R Core Team, 2013) using ez package (Lawrence, 2013), lme4 package (Bates et al., 2013), afex package (Singmann, 2013), and lmerTest package (Kuznetsova et al., 2013).

#### Dual task effect

The effect of dual task was assessed on success rate and DT. Overall time was not used because the dual task condition requires an additional time-consuming operation (i.e., reaching the shelter house) with respect to the diamond task.

**FIGURE 8:**
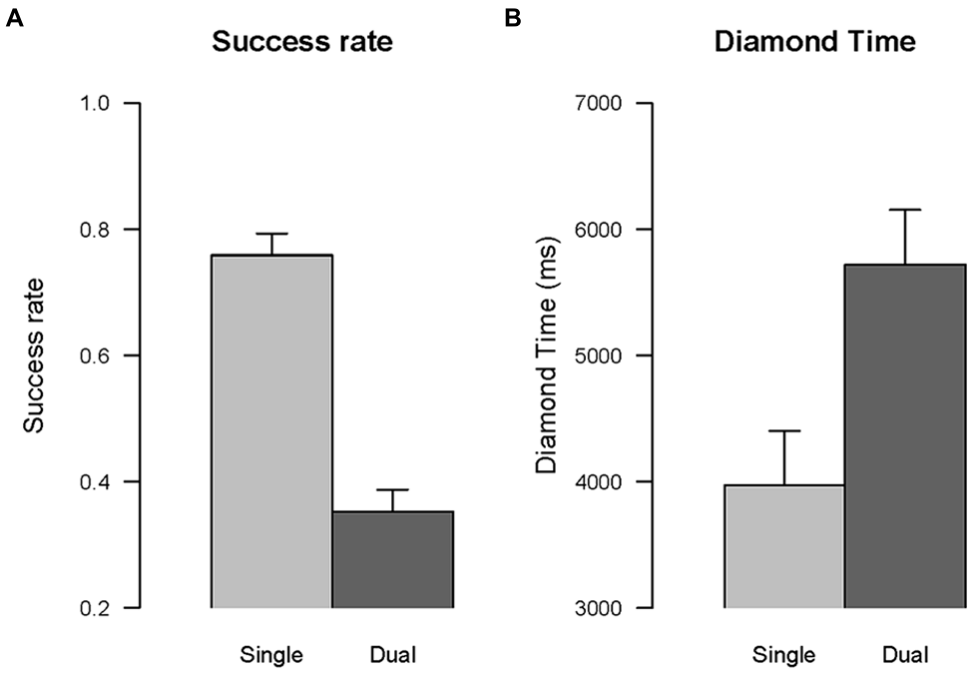
**Dual task effect.** The comparison between single task and dual task conditions is shown for success rate **(A)**, and time to collect the fist diamond **(B)**. Error bars are within-subjects confidence intervals calculated with the method of Morey (2008).

***Success rate.*** The effect of the type of task, single vs. dual, was significant, *F*(1,16) = 311.42, *p* < 0.001, $\eta_{G}^{2}$ = 0.91, indicating that in the dual task condition participants were less successful than in the single task condition (see **Figure 8A**). For example, the player was caught by the snake more often in the dual task than in the snake task, *F*(1,16) = 33.31, *p* < 0.05, $\eta_{G}^{2}$ = 0.46.

***Diamond time.*** The effect of the type of task, single vs. dual, was significant, *F*(1,16) = 36.21, *p* < 0.001, $\eta_{G}^{2}$ = 0.47, indicating that the time to collect the first diamond in the dual task condition was longer than in the single task condition (see **Figure 8B**).

#### Task switch effect

**FIGURE 9:**
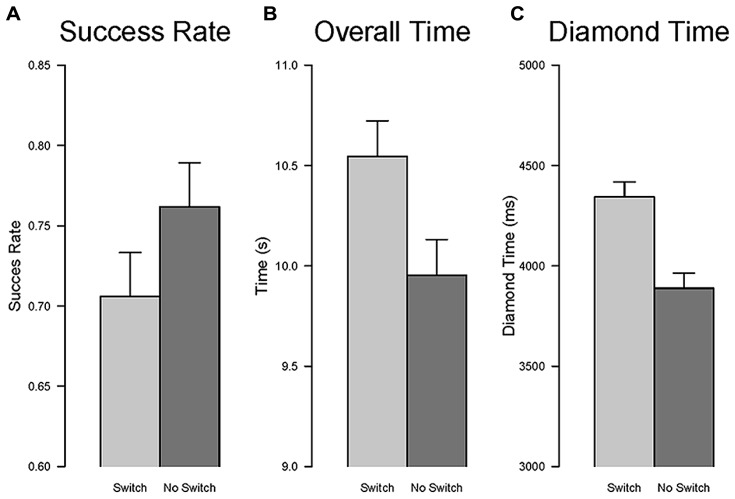
**Task switch effect.** The comparison between repetition and switch conditions is shown for success rate **(A)**, overall time to complete the task **(B)**, and time to collect the fist diamond **(C)**. Error bars are within-subjects confidence intervals calculated with the method of Morey (2008).

***Success rate.*** The effect of the type of task, new vs. repeated, was significant, *F*(1,16) = 9.35, *p* < 0.01, $\eta_{G}^{2}$ = 0.35, indicating that participants were less successful in trials involving a change of task relative to trials in which the task remained the same, that is a task switching cost (see **Figure 9A**).

***Overall time.*** The effect of the type of task, new vs. repeated, was significant, *F*(1,16) = 25.08, *p* < 0.001, $\eta_{G}^{2}$ = 0.09, indicating that participants were slower in completing the task for trials involving a change of task relative to trials in which the task remained the same (see **Figure 9B**).

***Diamond time.*** The effect of the type of task, new vs. repeated, was significant, *F*(1,16) = 83.11, *p* < 0.001, $\eta_{G}^{2}$ = 0.25, indicating that participants were slower to collect the first diamond for trials involving a change of task relative to trials in which the task remained the same (see **Figure 9C**).

#### Effect of training

We assessed the presence of a training effect within the game (i.e., performance improvement as a function of training time) in terms of changes in task difficulty level and time limit selected by the algorithm across the 14 sessions. Moreover, we assessed if the dual task and the task switching performance in the DT measure improved during the training. We employed mixed-effect multiple regression models (Baayen et al., 2008). By-subject random intercepts were included in all analyses. For the analyses of task difficulty and time limit we applied a logarithmic link function (Jaeger, 2008) and Poisson variance distribution that is appropriate for counts of events in a fixed time window (e.g., Baayen, 2008). For the DT analysis we performed Type III test calculating *p*-values via the likelihood ratio test in order to assess the significance of the main effects and the interactions of the predictors (i.e., session and condition).

***Task difficulty.*** The effect of the session was significant (*b* = 0.0021, *z* = 4.59, *p* < 0.001), indicating that the task difficulty increased (positive beta weight) across the sessions. In the last session, the participants reached a mean difficulty level of 4.67 (SD = 0.14).

***Time limit.*** The effect of the session was significant (*b* = -0.0040, *z* = -15.90, *p* < 0.001), indicating that the time limit decreased (negative beta weight) across the sessions. In the last session, the participants reached a mean time limit of 15.95 (SD = 0.62).

***Diamond time: dual task effect.*** The main effect of session was significant, χ^2^(1) = 135.71, *p* < 0.001, indicating that the time to collect the first diamond decreased across sessions. The main effect of condition (single vs. dual) was significant, χ^2^(1) = 749.41, *p* < 0.001, indicating that the DT in the dual task condition was longer than in the single task condition. The interaction session by condition was significant χ^2^(1) = 80.73, *p* < 0.001, indicating that the effect of the session was different for the two conditions. The interaction was inspected by changing the reference level accordingly with the desired contrast. The decrease in DT was significant for both conditions, but the reduction was larger for the dual task condition as attested by the larger (negative) beta weight (*b* = -8.19, *t* = -3.93, *p* < 0.001 and *b* = -63.35, *t* = -10.98, *p* < 0.001 for single and dual task conditions, respectively).

***Diamond time: task switch effect.*** The main effect of session was significant χ^2^(1) = 40.33, *p* < 0.001, indicating that the DT decreased across sessions. The main effect of condition (new vs. repeated) was significant χ^2^(1) = 105.98, *p* < 0.001, indicating that participants were slower to collect the first diamond for trials involving a change of task relative to trials in which the task remained the same. The interaction session by condition was significant χ^2^(1) = 9.45, *p* < 0.01, indicating that the effect of the session was different for the two conditions. The interaction was inspected by changing the reference level accordingly with the desired contrast. The decrease in DT was significant for both conditions, but the reduction was larger for the switch (new task) condition as attested by the larger (negative) beta weight (*b* = -6.79, *t* = -2.07, *p* < 0.05 and *b* = -19.56, *t* = -7.70, *p* < 0.001, for repeated and new conditions, respectively).

## DISCUSSION

The aim of this experiment was to validate the game “Labyrinth” in a study on unimpaired participants. Playing a game with these characteristics is likely to involve many different cognitive skills, some more basic, and some of a higher level. For example, successful playing requires selecting the relevant information and discarding the irrelevant ones. Playing until the end of the session requires to sustain attention at an adequate level for a relatively long time. Since the game was conceived to tap specific abilities, we first assessed whether playing the game involved these skills. In particular, we assessed whether the participants’ performance showed the cost of dual tasking and the cost of task switching to confirm the involvement of divided and alternate attention or flexibility.

The performance of the unimpaired participants in the first play session with the videogame showed the classic cost of dual task across the different performance measures. The success rate was higher in the single tasks than in the dual task condition. The dual task effect was confirmed also in the time dependent variable: the time to collect the first diamond was longer when the gamer had to collect the diamond and to avoid the snake at the same time compared to when she only had to collect diamonds. Therefore, the results confirm a robust dual task effect, thereby showing that completing the two tasks simultaneously requires to divide attention between the two goals (as well as between diamond and snake stimuli).

The analyses of the three performance measures also revealed a robust effect of task switching. In this case we compared the performance between the condition of repetition, when one task followed a task of the same type (e.g., DT after DT), with the condition of non-repetition, when one task followed a task of the other type (e.g., DT after ST). Success rate was higher in the condition of repetition than in the switch condition, in line with the findings using the classic task switch paradigm (Monsell, 2003). Likewise, the time to complete the task and the time to collect the first diamond showed a switch cost, with longer times for the switch condition compared to the repetition condition. Therefore, changing the task showed the need for reconfiguration or inhibition of the cognitive set of the prior task, thereby involving cognitive flexibility.

Overall, the performance improved throughout the training as indicated by the increase of task difficulty across sessions. This means that the algorithm moved the performance threshold toward more difficult levels because the participants became more skilled in the achievement of the goals. In the same vein, the maximum time allowed to accomplish the task decreased across sessions, indicating that participants became faster in the achievement of the goals. Moreover, using DT as performance index, we found that the cost of dual-tasking as well as the cost of task switching decreased during training. Though the time to collect the first diamond showed an overall decrease across sessions, the improvement was significantly stronger for the dual task condition than for the single task condition, thereby suggesting that players became more efficient in route planning under dual task. In the same vein, the comparison between repeated and new task conditions (i.e., task switching) showed a stronger performance improvement for the switch condition. These results suggest that playing with Labyrinth enhanced the participants’ attentional control, at least in terms of the ability to manage multitasking and to quickly reconfigure the task set. This finding is in line with studies showing that extensive dual task training enhances the ability of multitasking (Van Selst et al., 1999; Schumacher et al., 2001; Tombu and Jolicoeur, 2004).

The generalization beyond the task used for training is an important issue in the area of cognitive enhancement and rehabilitation. The training effect should transfer to other tasks to make the training really beneficial. We leave this issue to a follow-up study, but we believe that the characteristics of the game, for example the alternation between tasks as well as multitasking, may stimulate high levels attention functions as opposed to task specialization. Flexibility and control over attentional resources is clearly relevant in a variety of daily-life situations. An investigation of the relationship between videogame play and a comprehensive battery of cognitive / attentional tests would indeed clarify this issue (see Baniqued et al., 2013) and it would explicitly assess transfer to specific skills like task switching and multitasking.

## CONCLUSION

There is a growing body of evidence that videogame playing can enhance a variety of specific skills in addition to speeding up information processing (e.g., Hubert-Wallander et al., 2011a). Moreover, gaming seems to promote transfer to more ecological settings and generalization to untrained skills. Here we attempted to design a new videogame including specific features that were conceived to specifically involve attention and executive functions, with the final purpose to use it in supporting the rehabilitation practice of TBI patients. Cognitive deficits following TBI can profoundly affect daily living (Sohlberg and Mateer, 2001) because they often involve executive and attentional functions that are fundamental to control and modulate other more basic abilities. The design of the game was guided by principles relevant for the training of those functions. Therefore, its aim was to enhance mental flexibility (switching between different cognitive sets) and multi-tasking (maintain the cognitive sets of two different tasks and dividing attentional resources between two goals), stimulate planning ability (choosing the adequate strategy, interrupting automatic responses and monitoring performance), and encourage speeding up of processing. Most importantly, the videogame was equipped with a multidimensional adaptive algorithm that provided a continuous, online calibration of the level of difficulty across three different dimensions to the gamer’s current performance. We believe that this latter feature is crucial for managing the performance variability of patients. The development of the game included different testing stages. In the first stage, we simulated users with different performance profiles to assess the efficiency of the adaptive algorithm in estimating the user ability. In the second stage of the testing phase, we validated the game with unimpaired participants to ensure that the game involves the activation of the desired cognitive functions as well as to assess the effect of a short training period. Thus, the next step will be to test the videogame in a controlled clinical trial with TBI patients to assess if it is useful for the remediation of attentional and executive impairments.

## Conflict of Interest Statement

The authors declare that the research was conducted in the absence of any commercial or financial relationships that could be construed as a potential conflict of interest.
